# Sciatic nerve schwannoma complicated by nerve bundle membrane effusion: Two case reports and a literature review

**DOI:** 10.3389/fonc.2022.915982

**Published:** 2022-09-14

**Authors:** Lian He, Yan-Ji Zhang, Shi-Li He, Jia-Ren Zhang, Jun Wu, Gao-Feng Zhang, Da-Li Wang

**Affiliations:** ^1^ Department of Radiology, The Second Affiliated Hospital of Zunyi Medical University, Zunyi, China; ^2^ Department of Radiology, The First People’s Hospital of Zunyi, The Third Affiliated Hospital of Zunyi Medical University, Zunyi, China; ^3^ Department of Burn Plastic Surgery, The Affiliated Hospital of Zunyi Medical University, Zunyi, China

**Keywords:** schwannoma, MRI, case report, sciatic nerve, nerve bundle membranes effusion

## Abstract

Schwannoma is a benign tumor that originates from Schwann cells in the peripheral nerve tunica or bundle of nerves and grows along the longitudinal axis of the nerve. Schwannoma can occur in multiple anatomic locations but rarely in the sciatic nerve. To our knowledge, there are no previous reports in the literature related to schwannoma combined with effusion of the nerve bundle membranes. Here, we report two cases of sciatic nerve schwannoma combined with nerve bundle membrane effusion, and the relationship between them is uncertain. We have boldly speculated about this uncertain relationship by combining the two patients’ imaging manifestations to help determine the mechanism of schwannoma or effusion generation as well as a clinical treatment.

## Introduction

Schwannoma is one of the benign peripheral schwannomas, with an annual reported incidence of 0.3−0.4/100,000 people (1, 2). Schwannoma often occurs in the spinal nerve roots of the head and neck and the nerves on the flexion side of the limbs and occurs very rarely in the sciatic nerve, accounting for <1% of all sites (3). Depending on the anatomical site where the tumor appears, different clinical manifestations can then occur, mostly manifesting as sciatica, limb weakness, or lumbar disc herniation. In the previous literature, only the manifestation of nerve thickening has been mentioned, and no effusion of the nervous bundle membranes has been reported in the literature of the time being (4); the combination of sciatic nerve schwannoma with nerve bundle membrane effusion is even rarer. The imaging manifestations of this group of cases were analyzed in order to improve our understanding of schwannoma and to provide assistance in the diagnosis and treatment of this disease in clinical practice.

## Case reports

### Case 1

**Figure 1:**
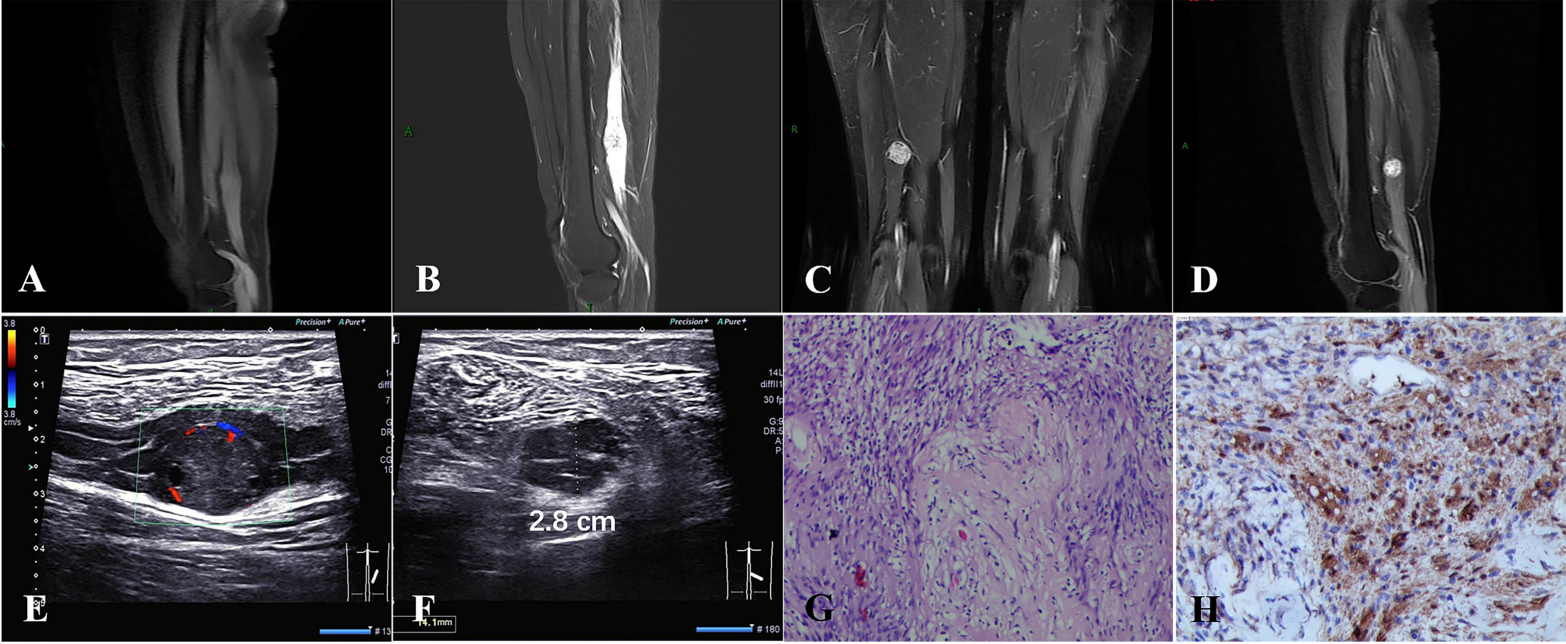
**(A–D)** A round iso-T1-long T2 signal nodule was located in the middle and lower third of the right sciatic nerve with significant enhancement. **(E, F)** A schwannoma of approximately 2.8 cm in diameter located in the sciatic nerve, with nerve bundle dilated with fluid and deformed by compression. **(G)** Hematoxylin and eosin staining showed schwannoma, Antoni A areas formed by closely packed tumor spindle cells, and loose mucus-like Antoni B areas, and no nerve fibers were visible in the tumor. **(H)** Immunohistochemistry showed that S-100 was scattered positive expression [original magnification **(G, H)**, ×100].

A 49-year-old female patient had progressive right lower extremity radiating pain for 2 months. Physical examination revealed a hard mass in the posterior calf with pressure and radiating pain and no abnormal muscle strength. Magnetic resonance imaging (MRI) showed a round-like nodule in the lower segment of the right sciatic nerve with iso-signal on T1-weighted images and hyper-signal on T2-weighted images with clear borders and the nerve trunk penetrating through it (**Figures 1A–C**). Contrast-enhanced examination showed significant enhancement of the tumor (**Figure 1D**). Ultrasound showed schwannoma located in the sciatic nerve (**Figure 1E**), with the nerve bundle dilated with fluid and deformed by compression (**Figure 1F**). There was no abnormality of the contralateral sciatic nerve. At subsequent surgery, the tumor was approximately 2.8 cm in diameter and was located between the nerve bundles. The nerve bundles were displaced bilaterally due to compression and thickening, and then the tumor was completely removed. Under the microscope, the nerve bundle membrane was incised longitudinally about 0.2 cm, the yellow nerve fibers were seen, and a small amount of clear fluid came out. Hematoxylin and eosin staining showed schwannoma and Antoni A areas formed by closely packed tumor spindle cells, and loose mucus-like Antoni B areas are seen; case 1 had no nerve fibers in the tumor (**Figure 1G**). Immunohistochemistry showed scattered positive expression of S-100 protein (**Figure 1H**). At 1-month postoperative follow-up, the pain in the right lower extremity was significantly reduced, and MRI showed no change in the effusion of the right sciatic nerve and no tumor residue.

### Case 2

**Figure 2:**
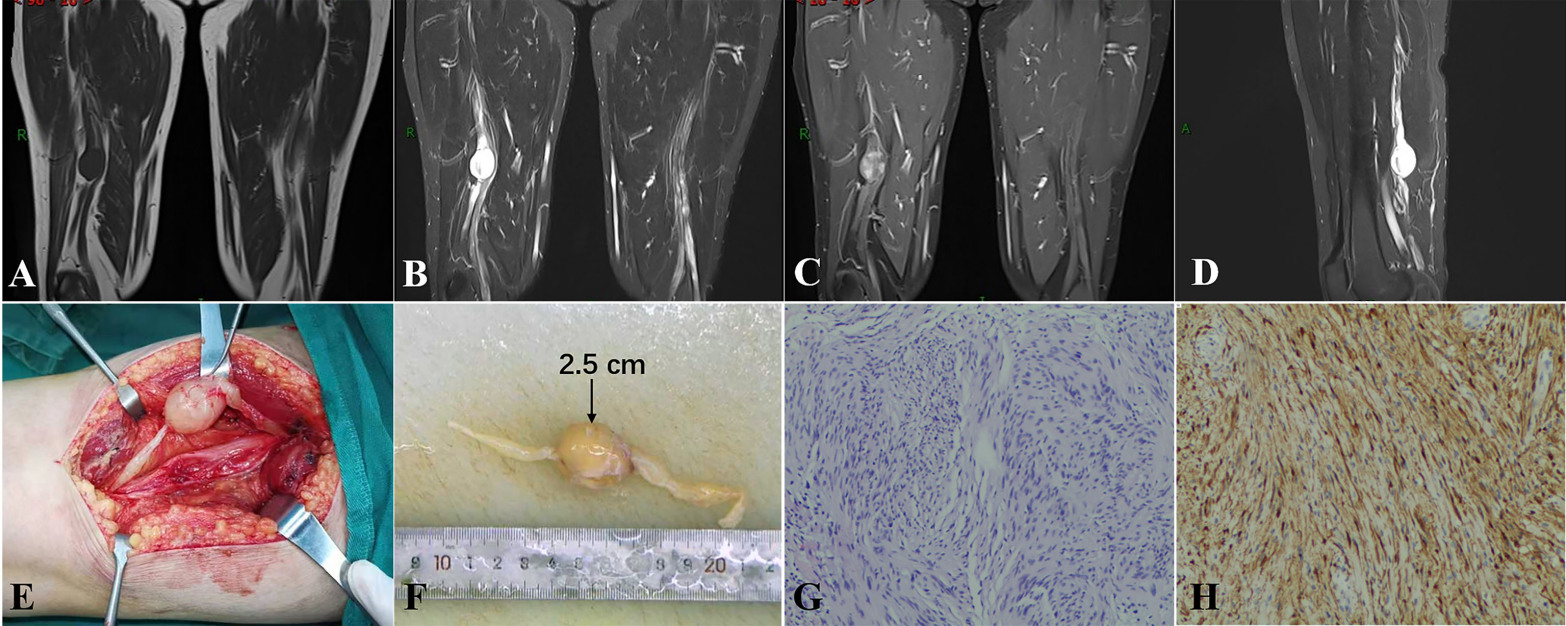
**(A–D)** A round-like iso-T1-long T2 signal nodule was located in the middle and lower third of the right sciatic nerve with progressive enhancement. The right sciatic nerve, tibial nerve, and common peroneal nerve trunk were extensively thickened, and the nerve bundles within them were thickened with a watery signal. **(E, F)** A gray-white nodule of 2.5-cm diameter with an intact envelope was seen on the incisional nerve epineurium. **(G)** Hematoxylin and eosin staining showed schwannoma, tumor spindle cells closely arranged in the Antoni A area, and nerve fibers visible in the tumor. **(H)** Immunohistochemistry showed scattered positive expression of S-100 protein [original magnification **(G, H)**, ×100].

A 45-year-old man had radiating pain in the right lower extremity for 4 years with a right thigh mass for 1 year. On physical examination, he had a hard nodule in the posterior part of the lower thigh with tenderness and radiating pain in the calf, atrophy of the right calf (+++), and muscle strength grade III. MRI showed a round-like nodule in the lower segment of the right sciatic nerve with iso-signal on T1-weighted images and hyper-signal on T2-weighted images with clear borders and the nerve trunk penetrating through it (**Figures 2A–C**). Contrast-enhanced examination showed progressive enhancement (**Figure 2D**). The right sciatic nerve, tibial nerve, and the common peroneal nerve trunk were extensively thickened with a diameter of approximately 0.5–0.7 cm in the two patients, the nerve bundles within them were thickened with a watery signal without enhancement, and the nerve fiber bundles were centrally located. The contralateral sciatic nerve bundle was thickened with a watery signal and no tumor. This patient subsequently underwent surgery. In the course of the operation, a marked thickening and limited enlargement of the right lower sciatic nerve were seen, and a grayish-white nodule with an intact envelope was seen on the incision of the nerve epineurium (**Figures 2E, F**). The tumor was 2.5 cm in diameter and located on one of the nerve bundles in which the nerve bundle penetrated and could not be separated. The tumor and its attached nerve bundle were completely excised, and the rest of the nerve bundles at the same time were thickened in a bead-like manner. Hematoxylin and eosin staining showed schwannoma, tumor spindle cells closely arranged in the Antoni A area, visible nerve fibers in the tumor, nerve bundle membrane, and a significantly enlarged endothelial gap (**Figure 2G**). Immunohistochemistry showed scattered positive expression of S-100 protein (**Figure 2H**). This patient refused follow-up.

## Discussion

Peripheral schwannoma originates from Schwann cells in the peripheral nerve outer membrane or nerve bundle membrane and is most common in the head, neck, and flexors of the extremities; it is prevalent in people aged 20–50 years with no gender differences (5, 6). The main mechanisms of schwannoma development include alteration of the axonal microenvironment and mechanical nerve stimulation (7). The main clinical manifestations are sciatica and lumbar disc herniation symptoms. The diagnosis of schwannoma is usually confirmed by MRI examination, which is mainly based on T1WI iso-signal T2WI high signal, and the T2WI signal will be higher when cystic necrosis occurs. Enhanced scans show significant heterogeneous enhancement in the solid part, and the Antoni A area, which is rich in tumor cells, is the main area of enhancement, while the Antoni B area is the main area of non-enhancement or weak enhancement. The tumors in this paper are mainly in the Antoni A region, and the Antoni B region is less commonly found, which we believe is the reason why the “target sign” did not appear (2). Most schwannomas are benign, and only a few become malignant (2).

Schwannomas often have an intact envelope and are pathologically composed of a multicellular Antoni A zone and a loose mucus-like Antoni B zone. The difference in the ratio of the two tissue conformations determines the difference in tumor MRI signal (8). A strongly positive expression of the S-100 protein was seen by immunohistochemistry (9). The majority of schwannomas of the thick nerve trunk occur between the nerve bundles under the epineurium, and a few occur in the nerve bundle membrane. Schwannomas under the epineurium appear early because of the symptoms of nerve compression, and the size of the tumor usually does not exceed 3 cm when found, which is mainly composed of the nerve epineurium and residual nerve fibers, with the nerve bundle being compressed around the tumor and the nerve fibers not passing through the tumor. The schwannoma in case 1 of this report occurred between the nerve bundle membranes under the epineurium of the sciatic nerve; the imaging pathology and clinical features were as described above. Tumors that originate from the nerve bundle are extremely rare; the tumor wraps around the nerve fibers and grows along the nerve, showing a nerve bundle access sign; case 2 is this type. Schwannoma, as reported in the literature, also has fat separation sign imaging features (10), which was not seen in the present two patients, and we speculate that the reason may be due to the location of the tumor under the outer membrane of the nerve stem and the small size of the tumor.

In our cases, an extremely rare common sign was found. The MRI presentation and surgery in both patients showed signs of a large effusion accumulation under the bundle membrane of the sciatic nerve on the affected side. Meanwhile, the sciatic nerve of the contralateral limb of case 2 not only had tumor growth but also showed signs of a bundle of effusion. Only the mechanism of spinal cystic schwannoma formation has been reported in the previous literature (11), but signs of sciatic schwannomas with nerve bundle membrane effusion have not been retrieved from the literature. The nerve bundle membrane is a component of the blood–nerve barrier and is essential to protect axons and their associated Schwann cells from infection by ion flow and toxins (12). There is no effusion between the normal nerve bundle and the endoneurium (13), and the causal relationship between the schwannoma of the sciatic nerve and effusion of the nerve bundle in this group of cases is uncertain. Neural bundle membrane glial cells make up the neural bundle membrane, which is a type of perineural glial cell and is essential for Schwann cell maturation and motor nerve assembly (14). Embryonic neural tract cells migrate from the spinal cord to form tubular structures that are accompanied by cerebrospinal fluid (14), which facilitates the growth of neuronal axons and prevents adhesion of the tracts to the endothelium. Under normal circumstances, the cerebrospinal fluid is absorbed when the nerve bundle matures. With the imaging presentation and surgical findings of this group of patients, we hypothesize that the patients first had nerve bundle membrane effusion due to the nerve bundle being affected by unfavorable factors during growth and the fluid is not absorbed, which then led to changes in the axonal microenvironment or mechanical stimulation of nerves and then induced schwannoma. The main basis is that there was no change in the nerve bundle with dilated effusion before and after tumor resection in case 1, and the contralateral sciatic nerve in case 2 had this sign even though there was no tumor. However, we still need more cases to prove this conjecture.

In conclusion, MRI examination has obvious advantages for the localization and qualitative diagnosis of peripheral large schwannoma, which also have important guiding value for the selection of treatment modalities for patients. The combination of sciatic nerve schwannoma and effusion of nerve bundle membranes is extremely rare, and more cases are needed to summarize the imaging and clinical features if we want to clarify the pathogenesis relationship between the two. This will enable clinicians to have a better understanding of schwannomas and to have earlier interventions as well as better treatments in the clinical setting.

## Data availability statement

The original contributions presented in the study are included in the article/supplementary material. Further inquiries can be directed to the corresponding authors.

## Ethics statement

Ethical review and approval was not required for the study on human participants in accordance with the local legislation and institutional requirements. The patients/participants provided their written informed consent to participate in this study. Written informed consent was obtained from the individual(s) for the publication of any potentially identifiable images or data included in this article.

## Author contributions

LH and Y-JZ contributed equally to the development of the manuscript. G-FZ and D-LW contributed equally and provided expert oversight for the completion of the manuscript. J-RZ provided images and modified them. S-LH and JW provided input on the preparation of the manuscript and later modified the article. All authors contributed to the article and approved the submitted version.

## Acknowledgments

We would like to thank Dr. Lei Gong for the revision of our article.

## Conflict of interest

The authors declare that the research was conducted in the absence of any commercial or financial relationships that could be construed as a potential conflict of interest.

## Publisher’s note

All claims expressed in this article are solely those of the authors and do not necessarily represent those of their affiliated organizations, or those of the publisher, the editors and the reviewers. Any product that may be evaluated in this article, or claim that may be made by its manufacturer, is not guaranteed or endorsed by the publisher.
